# Butylparaben Exposure Induced Darker Skin Pigmentation in Nile Tilapia (*Oreochromis niloticus*)

**DOI:** 10.3390/toxics11020119

**Published:** 2023-01-25

**Authors:** Song Liu, Nan Zhang, Zhifang Liang, Er-chao Li, Yong Wang, Shijie Zhang, Jiliang Zhang

**Affiliations:** 1 Ministry of Education Key Laboratory for Ecology of Tropical Islands, Key Laboratory of Tropical Animal and Plant Ecology of Hainan Province, College of Life Sciences, Hainan Normal University, Haikou 570100, China; 2 Hainan ForYou Ecological Environment Technology Co., Ltd., Haikou 570100, China; 3 School of Life Sciences, East China Normal University, Shanghai 200062, China

**Keywords:** parabens, neuroendocrine, phototransduction, melanophore, melanin

## Abstract

Butylparaben (BuP), as an emerging contaminant with endocrine-disrupting effects, may exert effects on skin pigmentation in fish by interfering with the neuroendocrine system. Therefore, models of BuP exposure in Nile tilapia (*Oreochromis niloticus*) were established by adding different doses of BuP (0, 5, 50, 500, and 5000 ng/L) for 56 days. The obtained results showed that BuP exposure induced darker skin pigmentation, manifested as increased melanin content of skin, while genes related to melanin synthesis, including *α-MSH* and *Asip2*, significantly changed. In addition, BuP exposure reduced dopamine and γ-aminobutyric acid content in the brain, which is related to the synthesis of *α-MSH*. Furthermore, the release of neurotransmitters from the brain is affected by light. Thus, the relative gene expression levels in the phototransduction pathway were evaluated to explore the molecular mechanism of BuP-induced darker skin pigmentation, and the obtained results showed that *Arr3a* and *Arr3b* expression was significantly upregulated, whereas *Opsin* expression was significantly downregulated in a BuP dose-dependent manner, indicating that BuP inhibited phototransduction from the retina to the brain. Importantly, correlation analysis results showed that all melanin indexes were significantly positively correlated with *Arr3b* expression and negatively correlated with *Opsin* expression. This study indicated that BuP induced darker skin pigmentation in Nile tilapia via the neuroendocrine circuit, which reveals the underlying molecular mechanism for the effects of contaminants in aquatic environments on skin pigmentation in fish.

## 1. Introduction

Skin pigmentation in fish plays an important role in camouflage, thermoregulation, photoprotection, mate choice, etc., but is also one of quality standards that dictates the market value for human consumption and ornamental use [1]. However, skin pigmentation is always influenced by numerous external factors, such as feed, tank coloration, light, and social interactions [2]. Among them, light, as one of the most important factors, exerts a direct effect on skin pigmentation by the primary color response and the secondary color response, which is involved in the neuroendocrine system [3]. For example, in white background adaptation, skin becomes pale, whereas skin darkens in black background adaptation, which is associated with α-melanocyte-stimulating hormone (*α-MSH*) levels regulated by the neuroendocrine circuit [4]. Evidently, photosensitivity in animals may be an indispensable factor in monitoring this shift. However, the underlying mechanism of neuroendocrine regulation of skin pigmentation in fish remains unclear.

Parabens are widely used in the cosmetics, food, and pharmaceutical industries for their broad antimicrobial activity and preservative properties, which inevitably lead to parabens entering the natural environment with increasing use [5]. Recently, growing evidence has shown that parabens, as endocrine-disrupting chemicals, can exert toxic effects on the skin [6], kidneys [7], liver [8], reproductive system [9], nerves [10], etc. In addition, endocrine-disrupting effects of parabens increased as the side-chain length increased from one to four [11]. Hence, paraben exposure may exert effects on skin pigmentation in fish by interfering with the neuroendocrine system.

Nile tilapia (*Oreochromis niloticus*) is an excellent model for toxicology research due to its short breeding cycle, simple treatment, and rapid response to environmental changes [12,13]. Furthermore, numerous color mutants in Nile tilapia can be obtained using natural and artificially induced methods, which makes it an outstanding model for studying the mechanisms of skin pigmentation changes [13]. 

In this study, different doses of butylparaben (BuP) were chosen according to previous studies [14,15,16] to evaluate the effects of BuP on skin pigmentation in Nile tilapia. Skin pigmentation-related hormones, neurotransmitter levels, and genes related to phototransduction were measured to reveal the underlying mechanisms involved in BuP-induced abnormal skin pigmentation by interfering with the neuroendocrine system.

## 2. Materials and Methods

### 2.1. Chemicals and Exposure

BuP (CAS number: 94-26-8, purity >99%) was purchased from Macklin Biochemical Co., Ltd. (Shanghai, China), and diluted with dimethyl sulfoxide (DMSO) to obtain 0.75, 7.5, 75, and 750 μg/mL BuP stock solutions. The stock solutions (200 μL) were directly spiked into each tank with 30 L of water to achieve final BuP concentrations of 5, 50, 500, and 5000 ng/L and named 5 ng/L BuP group, 50 ng/L BuP group, 500 ng/L BuP group, and 5000 ng/L BuP group, respectively. The final concentrations of DMSO were 0.0006% in each tank, and the control was only treated with 0.0006% DMSO, and named control group.

### 2.2. Fish Maintenance and Sampling

Nile tilapia were purchased from a local store (Lingao, Hainan, China), and then reared in experimental glass tanks containing filtered and chlorinated tap water. After two weeks of acclimation, the fish were randomly divided into five groups (three replicates per treatment group and twenty fish per replicate). Throughout the experiment, the fish were fed commercial feed (Tongwei Co., Ltd., Chengdu, China) at 9:00 and 17:00, the water was maintained at a pH 7.2 ± 0.2, a temperature of 30 ± 2.0 °C, and with 7.0 ± 0.2 mg/L dissolved oxygen, and the photoperiod was set at 12 h light:12 h dark. To maintain the original exposure concentration, 80% of the water in each tank was renewed daily. According to our previously reported methods [15], actual BuP concentrations (*n* = 3) in the control group and BuP exposure groups were measured each week using an LC/MS/MS system connected to an AB SCIEX QTRAP 5500 tandem quadrupole mass spectrometer (QTRAP^®^ 5500, Mundelein, IL, USA). The actual concentrations of BuP were 0, 5.19 ± 0.12, 50.26 ± 0.46, 518.33 ± 5.67, and 5286.67 ± 303.33 ng/L in control, 5 ng/L BuP, 50 ng/L BuP, 500 ng/L BuP, and 5000 ng/L BuP groups.

On the 56th day of BuP exposure, the fish were anesthetized using 20 mg/L MS-222 (Sigma, Osterode am Harz, Germany), placed on a white plate, and photographed using a camera (Snoy Alpha 7R III, Tokyo, Japan). The grey value analysis of skin (*n* = 5) was performed using ImageJ software (1.8.0, USA). Pictures were taken for background correction, and then the black area was separated by outlining using the wand tool, which was divided by the total lateral area of fish to count the relative area of dark skin. Subsequently, black striped skin tissue samples were collected using forceps after removing the scales. A part of the collected skin tissues was rapidly fixed with 4% paraformaldehyde for histology observation. The other part skin, eye, and, brain tissues were immediately frozen with liquid nitrogen, and then stored at −80 °C for future enzyme-linked immunosorbent assay (ELISA) and quantitative real-time PCR (RT-qPCR) analysis. All procedures involving animals were reviewed and approved by the Institutional Animal Care and Use Committee at Hainan Normal University.

### 2.3. Histology Analysis

After being fixed for 24 h in 4% paraformaldehyde, skin tissues were dehydrated in ethanol solution with increasing concentration, dealcoholized by xylene, embedded in paraffin, and sliced into 5 μm thick slices. Subsequently, the obtained skin sections were stained with hematoxylin and eosin and observed under a microscope (Nikon Ci-L, Tokyo, Japan). The relative area of melanin in skin tissues (*n* = 5) was analyzed by ImageJ software. Black linear or granular areas were defined as melanin, which was divided by the area of photographs (the enlarged images in Figure 2A) to enumerate the relative area of melanin in skin tissues.

### 2.4. ELISA

The eye and brain tissues (*n* = 5) were homogenized in ice-cold normal saline and then centrifuged at 3000 rpm for 10 min at 4 °C. Afterwards, the supernatants were used to determine the activity of tyrosinase (Tyr) and the content of melanin in the skins and dopamine (DA), γ-aminobutyric acid (GABA), neuropeptide Y (NPY), and acetylcholine (ACH) content in the brain according to the instructions of ELISA kits (Lai Er Bio-Tech, Hefei, China).

### 2.5. RT-qPCR Analysis

Total RNA in the eye and brain tissues (*n* = 5) was extracted according to the instructions of the TRIzol^®^ Reagent Kit (Invitrogen, Waltham, MA, USA). cDNA synthesis was conducted using Hiscript^®^ Q RT SuperMiX (Vazyme Biotech Co., Ltd., Nanjing, China). RT-qPCR analysis was performed on a CFX96Touch Real-time PCR Detection System (Bio-Rad, CA, USA) with ChamQ SYBR qPCR Master Mix (Vazyme Biotech Co., Ltd., Nanjing, China). The primers for *β-actin*, *α-MSH*, agouti signaling protein 2 (*Asip2*), rhodopsin (*Rh*), *Opsin*, phosphodiesterase (*PDE*), arrestin 3a (*Arr3a*), arrestin 3b (*Arr3b*), and recoverin (*Rec*) were designed using Primer 5.0 software (Table S1). The *β-actin* housekeeping gene served as the control. Following a previous study, relative expression levels were analyzed in this study using the 2^−ΔΔ^Ct method [17].

### 2.6. Statistical Analysis

All experimental data are expressed as the mean ± standard error (S.E.) and were analyzed for normality (Kolmogorov–Smirnov test) and variance homogeneity (Levene’s test). The data that did not meet above conditions was processed logarithmically. Significant differences (*p* < 0.05) between groups were evaluated using one-way analysis of variance (ANOVA) followed by Tukey’s post-hoc test or a non-parametric Kruskal–Wallis test in SPSS software (Version 13.0; Inc., Chicago, IL, USA). Spearman’s rank correlation was carried out to assess the correlation of melanin indexes with ELISA/Genes indexes in SPSS software. Coefficients close to 1 or −1 were strongly correlated.

## 3. Results

### 3.1. Melanin Content

**Figure 1:**
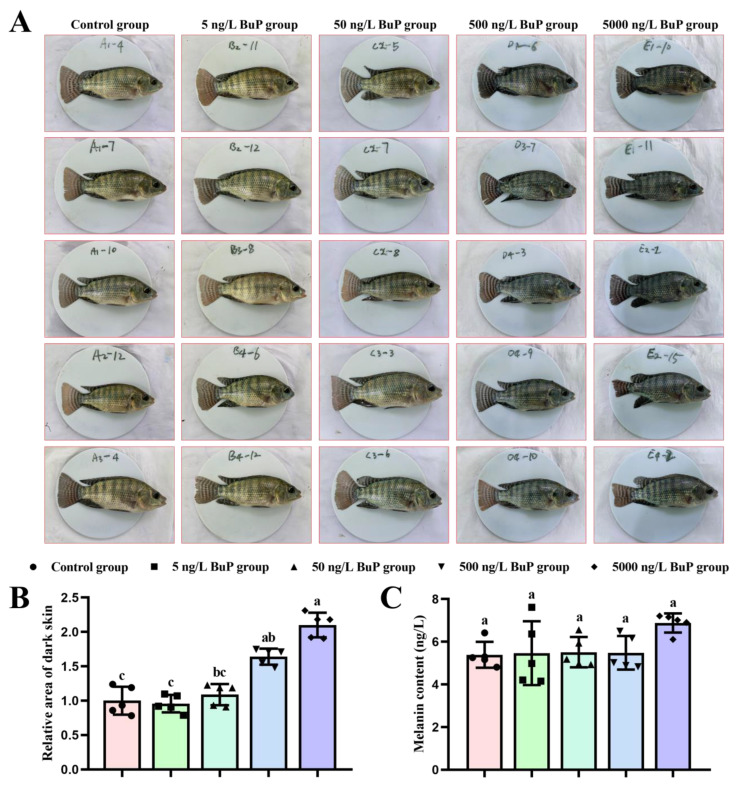
Effects of BuP on the melanin content in skin tissues. The pictures of fish (**A**); relative area of dark skin (**B**); and melanin content of the skin (**C**). Means of groups not sharing a common letter are significantly different at *p* < 0.05.

To investigate the effects of BuP on skin pigmentation of melanin in Nile tilapia, first the grey value of whole skin was photographed and analyzed (Figure 1). The stripes on the belly and caudal fin and the surrounding skin darkened after BuP treatment (Figure 1A), especially in the 500 ng/L BuP group and 5000 ng/L BuP group. Compared with the control group, the relative area of dark skin did not significantly change in the 5 and 50 ng/L BuP groups, but significantly increased in the 500 and 5000 ng/L BuP groups (Figure 1B). At the same time, the melanin content of skin was quantitatively analyzed by ELISA (Figure 1C), and the obtained results showed that when compared with the control group, the melanin content increased in the 5000 ng/L BuP group, but the difference was not significant. 

**Figure 2:**
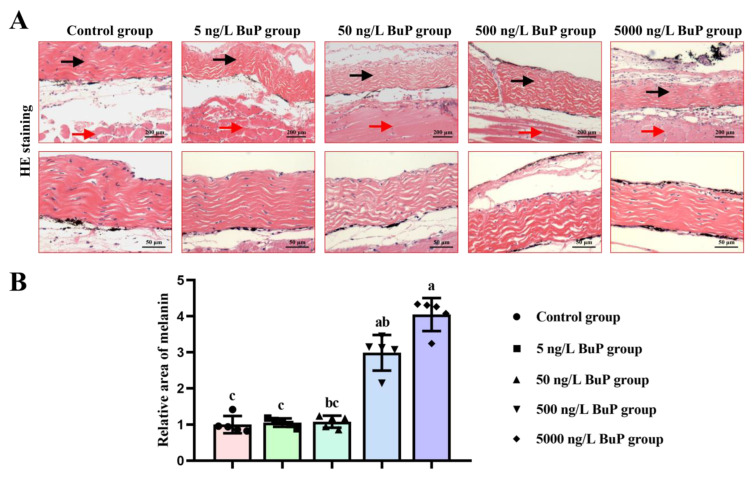
Effects of BuP on the morphological structures in skin tissues. HE staining (**A**); relative area of melanin (**B**). Black arrows indicate the dermis; red arrows show the muscle layer. Means of groups not sharing a common letter are significantly different at *p* < 0.05.

HE staining results showed that melanin was distributed in the dermis (Figure 2A). Moreover, melanin was mainly distributed on one side of the dermis in the control, 5 ng/L BuP, and 50 ng/L BuP groups. With an increase in BuP dose, melanin was distributed on both sides of the dermis in the 500 ng/L BuP group and 5000 ng/L BuP group. The relative area of melanin was significantly increased in a dose-dependent manner after BuP treatment (Figure 2B), particularly in the 500 and 5000 ng/L BuP groups, which confirmed the results in Figure 1. In summary, 500 and 5000 ng/L BuP induced darker skin pigmentation in Nile tilapia.

### 3.2. Enzymes and Genes Related to Melanin Synthesis

**Figure 3:**
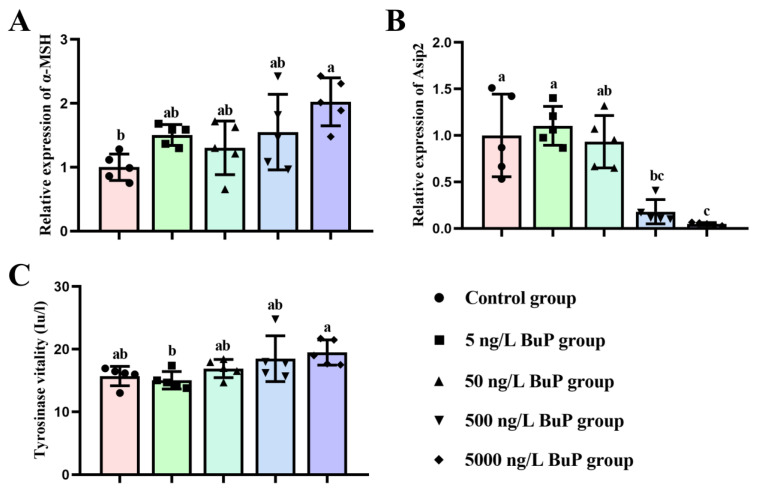
Effects of BuP on the expression levels of *α-MSH* (**A**) and *Asip2* (**B**) in the brain tissues, and the activity of Tyr (**C**) in the skin tissues. Means of groups not sharing a common letter are significantly different at *p* < 0.05.

As shown in Figure 3A, the relative expression level of *α-MSH* was significantly increased in the 5000 ng/L BuP group (2.024-fold; *p* = 0.003) compared with the control group. In addition, treatment with BuP significantly reduced the relative expression level of *Asip2* in the 500 ng/L BuP group (0.820-fold; *p* = 0.035) and the 5000 ng/L BuP group (0.953-fold; *p* = 0.001; Figure 3B). The ELISA results showed that exposure to BuP increased the activity of Tyr; however, the difference was not significant (Figure 3C). In addition, compared to the 5 ng/L BuP group, the activity of Tyr was significantly increased in the 5000 ng/L BuP group.

### 3.3. Neurotransmitter Content

**Figure 4:**
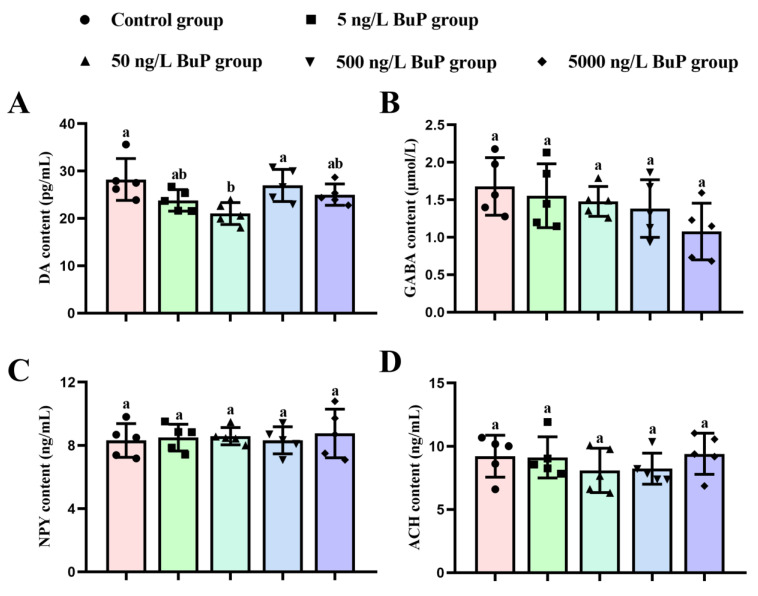
Effects of BuP on the content of DA (**A**), GABA (**B**), NPY (**C**), and ACH (**D**) in the brain tissues. Means of groups not sharing a common letter are significantly different at *p* < 0.05.

The measurement results of neurotransmitter contents in the brain tissues are shown in Figure 4. A significant reduction in the DA content was found only in the 50 ng/L BuP group compared with the control and 500 ng/L BuP groups (Figure 4A). However, compared with the control group, no significant changes in GABA (Figure 4B), NPY (Figure 4C), or ACH content (Figure 4D) were found in the BuP treatment groups.

### 3.4. Relative Gene Expression Levels in the Phototransduction Pathway

**Figure 5:**
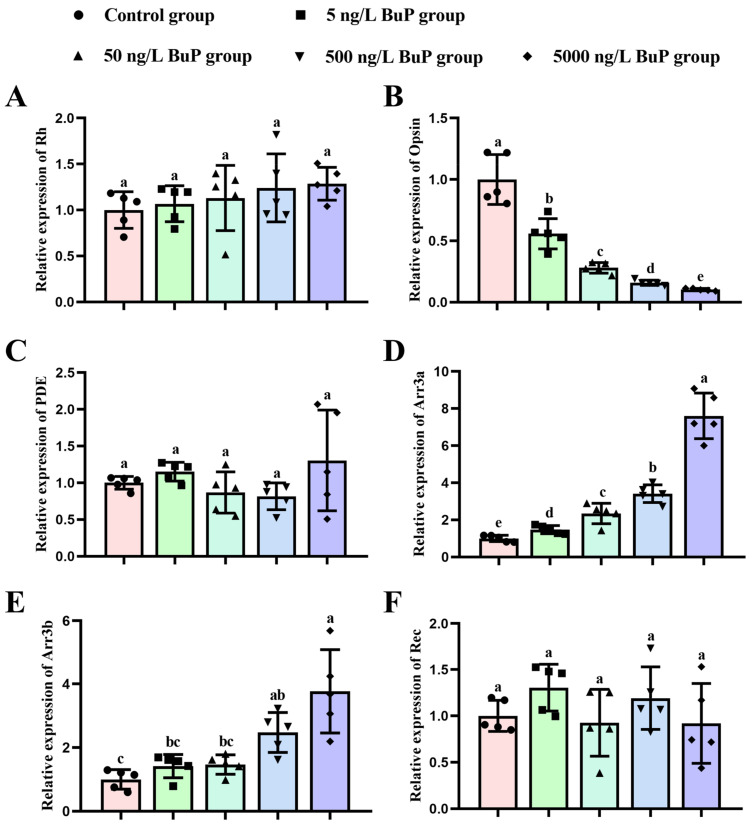
Effects of BuP on the relative expression levels of *Rh* (**A**), *Opsin* (**B**), *PDE* (**C**), *Arr3a* (**D**), *Arr3b* (**E**), and *Rec* (**F**) in eye tissues. Means of groups not sharing a common letter are significantly different at *p* < 0.05.

To explore the molecular mechanism of BuP-induced darker skin pigmentation, the relative gene expression levels in the phototransduction pathway, including *Rh*, *Opsin*, *Arr3a*, *Arr3b*, *Rec*, and *PDE*, were detected (Figure 5). The obtained results showed that exposure to BuP did not significantly affect *Rh* (Figure 5A), *PDE* (Figure 5C), or *Rec* expression (Figure 5F). However, the relative expression of *Opsin* was significantly reduced in a dose-dependent manner, especially in the 50 ng/L BuP group (0.720-fold; *p* = 0.000), the 500 ng/L BuP group (0.842-fold; *p* = 0.000), and the 5000 ng/L BuP group (0.897-fold; *p* = 0.000) compared with the control group (Figure 5B). In addition, BuP led to a significant increase in *Arr3a* (Figure 5D) and *Arr3a* expression (Figure 5E) with increasing BuP concentration, especially in the 500 ng/L BuP group (3.414-fold and 2.476-fold, respectively; *p* = 0.000 and *p* = 0.001, respectively) and the 5000 ng/L BuP group (7.607-fold and 3.774-fold, respectively; *p* = 0.000 and *p* = 0.000, respectively).

### 3.5. Correlation Analysis

As shown in Table S2, the relative area of dark skin was significantly positively correlated with Tyr content (*r* = 0.510, *p* = 0.009) and *α-MSH* (*r* = 0.445, *p* = 0.026), *Arr3a* (*r* = 0.787, *p* = 0.000), and *Arr3b* expression (*r* = 0.700, *p* = 0.000) levels; it was significantly negatively correlated with GABA content (*r* = −0.452, *p* = 0.023) and *Asip2* (*r* = −0.789, *p* = 0.000) and *Opsin* expression (*r* = −0.813, *p* = 0.000) levels. Melanin content was significantly positively correlated with the *Arr3b* expression (*r* = 0.422, *p* = 0.036) level and negatively correlated with the *Opsin* expression (*r* = −0.452, *p* = 0.023) level. The relative area of melanin was significantly positively correlated with Tyr content (*r* = 0.521, *p* = 0.008) and *α-MSH* (*r* = 0.510, *p* = 0.009), *Arr3a* (*r* = 0.778, *p* = 0.000), and *Arr3b* expression (*r* = 0.733, *p* = 0.000) levels; it was negatively correlated with GABA content (*r* = −0.455, *p* = 0.022) and *Asip2* (*r* = −0.774, *p* = 0.000) and *Opsin* expression (*r* = −0.805, *p* = 0.000) levels.

## 4. Discussion

Skin pigmentation is a manifestation of self-protection for fish to adapt to the environment during evolution; however, numerous pollutants have been confirmed to induce skin pigmentation abnormalities [18,19,20]. BuP, as an emerging contaminant with endocrine-disrupting effects, can exert toxic effects on multiple tissues and organs after long-term exposure. Studies have shown that the neuroendocrine circuit regulates skin pigmentation and is involved in phototransduction, neurotransmitter secretion, and melanin synthesis [4,21]. Therefore, we speculate that BuP interferes with this process and induces abnormal skin pigmentation in Nile tilapia. In this study, increased melanin content after BuP exposure was detected by various approaches. However, quantitative determination of melanin levels by ELISA showed that 5000 ng/L BuP treatment did not significantly increase melanin levels. We speculate that this occurred because black striped skin tissues were used to evaluate melanin levels in this study, but BuP mainly induces the darkening of surrounding skin. In addition, the *α-MSH* expression level was significantly upregulated, and the *Asip2* expression level was significantly upregulated. *α-MSH* secreted by the pituitary binds to the melanocortin 1 receptor (MC1R) on the skin and activates Tyr through a series of cascade reactions, thus promoting the synthesis of melanin [1]. In contrast, *Asip2*, as an *α-MSH* competitive antagonist of the MC1R, can inhibit the synthesis of melanin [22]. These results indicated that BuP exposure induced darker skin pigmentation.

Increasing evidence has confirmed that *α-MSH* expression levels are closely related to neurotransmitter contents; for example, suprachiasmatic melanotrope inhibitory neurons (SMIN) can release NPY, DA, and GABA, all of which can inhibit melanotropes in the pituitary to synthesize *a-MSH* in a synaptic way [4,23,24]. In this study, BuP reduced DA and GABA levels in the brain; however, the difference was not significant, which may be because neurotransmitters are efficient, and extremely low levels can alter this physiological process. Interestingly, a previous study showed that ACH can serve as a stimulator of *a-MSH* synthesis [25]; however, in this study, ACH content even decreased after BuP treatment, and the cause needs to be studied in the future.

The neurotransmitters released by SMIN are regulated by light, and when eyes were removed, the a-MSH levels increased and skin darkened [26,27]. Thus, the relative expression of genes in the phototransduction pathway was detected to uncover the underlying molecular mechanism of BuP-induced abnormal skin pigmentation. In most living animals, photoreceptors are located in the retina, and *Rh* composed of *Opsin* and 11-cis-retinal is a crucial photoactive substance in the retina [28]. When *Rh* captures photons, the 11-cis-retinal isomerizes and separates from *Opsin*, which produces Rh*, an active state of *Rh* [29,30]. Subsequently, Rh* binds to G-protein transducin and activates *PDE*, reducing cGMP concentration [31]. Ultimately, cGMP level reduction causes cGMP-gated Na^+^/Ca^2+^ channel closure and hyperpolarization of the photoreceptor, thus resulting in light signals being converted into electrical signals from the retina to the brain [32]. In this process, Rh* is regulated by *Rec* and *Arr*. On the one hand, Rh* can be inactivated by the phosphorylation of G-protein receptor kinase (Grk1) and subsequent binding to *Arr* [33]. On the other hand, *Rec*, as a negative regulator of Grk1, can inhibit the phosphorylation of Rh* [34]. In this study, the relative expression levels of *Arr3a* and *Arr3b* were significantly upregulated in a dose-dependent manner, indicating that BuP inhibited the activation of *Rh*, thus weakening phototransduction from the retina to the brain. In addition, *Rh* expression level was increased, and *Opsin* expression level was significantly reduced. This occurred because BuP exposure led to increased Rh* to restore the inactive state; thus, changes in *Rh* and *Opsin* expression levels were observed. Importantly, correlation analysis results showed that all melanin indexes were significantly positively correlated with *Arr3b* expression and negatively correlated with *Opsin* expression. Changes in the expression levels of these genes may be the key cause of BuP-induced darker skin pigmentation.

**Figure 6:**
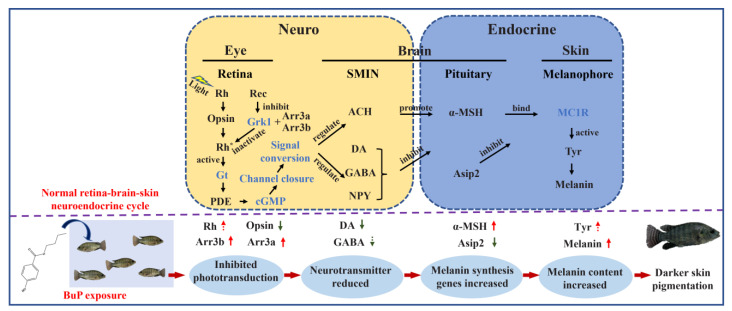
Schematic overview of underlying molecular mechanism of BuP-induced darker skin pigmentation in Nile tilapia.

In conclusion, as shown in Figure 6, BuP interferes with the gene expression levels in the phototransduction pathway, inhibiting phototransduction from the retina to the brain and reducing DA and GABA contents in the brain, thus disrupting the balance between *α-MSH* and *Asip2* and ultimately leading to darker skin pigmentation. This study reveals the underlying molecular mechanism of BuP-induced abnormal skin pigmentation in Nile tilapia. Given the importance of skin pigmentation for fish, further attention should be given to the toxic effects of contaminants in aquatic environments on skin pigmentation in fish.

## Data Availability

Not applicable.

## Supplementary Materials

The following supporting information can be downloaded at: https://www.mdpi.com/article/10.3390/toxics11020119/s1, Table S1: Sequence of primers used in RT-qPCR; Table S2: Correlation analysis of melanin indexes with ELISA/Genes indexes.
