# Supplementary material for: Butylparaben Exposure Induced Darker Skin Pigmentation in Nile Tilapia (Oreochromis niloticus)
Source: Toxics. 2023 Jan 25;11(2):119. doi: 10.3390/toxics11020119 (PMC9959106; doi:10.3390/toxics11020119)
Supplement: Supplementary file 1 [file toxics-11-00119-s001.zip › toxics-2135857-supplementary.pdf]

toxics

MDPI

Supplementary Materials: Butylparaben Exposure Induced Darker Skin Pigmentation in Nile Tilapia (*Oreochromis niloticus*)

Song Liu, Nan Zhang, Zhi-fang Liang, Er-chao Li, Yong Wang, Shi-jie Zhang and Ji-liang Zhang *

Table S1. Sequence of primers used in RT-qPCR

Gene symbol	Primer	Primer Sequence (5′-3′)
β-actin	Forward	TGGCTGAAGGCTACGTCATC
	Reverse	ACACATAGCTGAACCGCTCC
α-MSH	Forward	AGGAACCACAGCAAGAGCAG
	Reverse	GCTGTCCATCAGCTCCAGTT
Asip2	Forward	GTGAGGCCCAATCCTGTGAA
	Reverse	CATTGAAGAAGCGGCAGTGG
Rh	Forward	TCCCTGATGGAGGACCAGTT
	Reverse	ACGTACACCAGCTTGAAGGG
Opsin	Forward	ATGCACAGGTGGTGGACTTC
	Reverse	GAGGCATCGTTTGGGTCCTT
PDE	Forward	GGGCATCACAGTGCTCAATC
	Reverse	AGGATGAGTGAAAGCACCCAG
Arr3a	Forward	CGGAGAGACGGTGGACTCTA
	Reverse	GTGTCCTCGTCCTTCAGTCG
Arr3b	Forward	TTGCGGTGTGGACTTTGAGA
	Reverse	GTTAGCTGGGGCGAACTGTA
Rec	Forward	TCATGGCTGGAATGACGGAT
	Reverse	AGGCTTTCTCCAGACGAGAC

Table S2. Correlation analysis of melanin indexes with ELISA/Genes indexes.

Melanin Indexes	ELISA/Genes Indexes	*r*	*p*-value
Relative area of dark skin	*α-MSH*	0.445	0.026
Relative area of dark skin	*Asip2*	−0.789	0.000
Relative area of dark skin	Tyr	0.510	0.009
Relative area of dark skin	DA	0.125	0.550
Relative area of dark skin	GABA	−0.452	0.023
Relative area of dark skin	NPY	−0.093	0.658
Relative area of dark skin	ACH	0.032	0.878
Relative area of dark skin	*Rh*	0.277	0.179
Relative area of dark skin	*Opsin*	−0.813	0.000
Relative area of dark skin	*PDE*	−0.123	0.558
Relative area of dark skin	*Arr3a*	0.787	0.000
Relative area of dark skin	*Arr3b*	0.700	0.000
Relative area of dark skin	*Rec*	−0.143	0.495
Melanin content	*α-MSH*	0.038	0.857
Melanin content	*Asip2*	−0.365	0.072
Melanin content	Tyr	0.362	0.076
Melanin content	DA	0.029	0.890
Melanin content	GABA	−0.348	0.088
Melanin content	NPY	0.122	0.560
Melanin content	ACH	0.217	0.298
Melanin content	*Rh*	0.064	0.760
Melanin content	*Opsin*	−0.452	0.023
Melanin content	*PDE*	0.078	0.709
Melanin content	*Arr3a*	0.344	0.092
Melanin content	*Arr3b*	0.422	0.036
Melanin content	*Rec*	−0.185	0.376
Relative area of melanin	*α-MSH*	0.510	0.009
Relative area of melanin	*Asip2*	−0.774	0.000
Relative area of melanin	Tyr	0.521	0.008
Relative area of melanin	DA	0.127	0.545
Relative area of melanin	GABA	−0.455	0.022
Relative area of melanin	NPY	−0.077	0.715
Relative area of melanin	ACH	−0.028	0.893
Relative area of melanin	*Rh*	0.278	0.179
Relative area of melanin	*Opsin*	−0.805	0.000
Relative area of melanin	*PDE*	−0.137	0.515
Relative area of melanin	*Arr3a*	0.778	0.000
Relative area of melanin	*Arr3b*	0.733	0.000
Relative area of melanin	*Rec*	−0.157	0.453
